# Monoclonal antibodies in idiopathic chronic eosinophilic pneumonia: a scoping review

**DOI:** 10.1186/s12890-024-02868-3

**Published:** 2024-02-08

**Authors:** Andrea Dionelly Murillo, Ana Isabel Castrillon, Carlos Daniel Serrano, Liliana Fernandez-Trujillo

**Affiliations:** 1 Department of Internal Medicine, Allergology Service, Fundación Valle del Lili, Carrera 98 # 18-49, Cali, 760032 Colombia; 2 Faculty of Health Sciences, Universidad Icesi, Calle 18 # 122-135, Cali, 760032 Colombia; 3 Clinical Research Center, Fundación Valle del Lili, Carrera 98 # 18-49, Cali, 760032 Colombia; 4 Department of Internal Medicine, Pulmonology Service, Interventional Pulmonology. Fundacion Valle del Lili, Av. Simón Bolívar. Carrera 98 # 18-49. Torre 6, 4th Floor, Cali, Colombia

**Keywords:** Carrington syndrome, Pulmonary eosinophilia, Eosinophilic pneumonia, Idiopathic chronic eosinophilic pneumonia, Systemic corticosteroids, Monoclonal antibodies

## Abstract

**Background:**

Idiopathic chronic eosinophilic pneumonia (ICEP) is a rare disease characterized by pulmonary radiological alterations, peripheral eosinophilia, and demonstrated pulmonary eosinophilia. Oral steroids (OSs) are the standard management, but relapses occur in up to 50% of patients during the decrease or suspension of steroids, usually requiring reinitiation of treatment, exposing patients to secondary events derived from the management. Management with monoclonal antibodies has been proposed in these cases to control the disease and limit the secondary effects. The objective is to describe the extent and type of evidence regarding the use of monoclonal antibodies for ICEP.

**Methods:**

A panoramic review of the literature was performed. Observational and experimental studies of pediatric and adult populations that managed recurrent ICEP with monoclonal antibodies were included. Data search, selection, and extraction were performed by two independent reviewers.

**Results:**

937 studies were found. After applying the inclusion and exclusion criteria, 37 titles remained for the final analysis: a retrospective, observational, real-life study, two case series publications, and 34 case reports published in academic poster sessions and letters to the editor. In general, the use of monoclonal antibodies approved for severe asthma could be useful for the control of ICEP, since most of the results show a good response for clinical and radiological outcomes. Biological drugs seem to be a safer option for controlling relapses in ICEP, allowing lowering/suspension of OSs, and sometimes replacing them in patients intolerant to them, patients with significant comorbidities, and patients who have already developed adverse events.

**Conclusion:**

The extent of the evidence supporting management of ICEP with monoclonal antibodies against IL-5 and IgE (omalizumab) is limited, but it could be promising in patients who present frequent relapses, in cortico-dependent individuals, or in patients in whom the use of steroids is contraindicated. The extent of the evidence for management with dupilumab is more limited. Studies with better design and structure are needed to evaluate quality of life and outcomes during a clear follow-up period. To our knowledge, this is the first scoping review of the literature showing the extent of the evidence for the management of ICEP with monoclonal antibodies.

## Background

Eosinophilic pulmonary diseases comprise a rare and heterogeneous group characterized by eosinophilic infiltrate in the lung parenchyma as found in bronchoalveolar lavage (BAL) (generally at a proportion > 25%) or by biopsy. In most cases, there is peripheral eosinophilia (> 500 × 10 cells/L) [1, 2]. According to the existence of a specific cause, it is classified as primary or secondary. The etiology is broad and includes fungal and parasitic infections, drugs, toxins, autoimmune inflammatory diseases, and neoplasms, among others. When there is no evidence of underlying disease, it is classified as primary or idiopathic, so this is diagnosed by exclusion [1–3].

Eosinophilic pneumonia is a primary disorder that can be acute or chronic. Acute eosinophilic pneumonia has an acute, rapidly progressive presentation with fever, severe hypoxemia, and respiratory failure [2, 4]. It is more common in adults, affecting men most often. Generally, there is no history of asthma. It responds strongly to systemic steroids, without relapses and with resolution of the acute event [2, 4, 5].

Idiopathic chronic eosinophilic pneumonia (ICEP), or Carrington syndrome, is a rare disease with unknown prevalence. It has a subacute presentation, with chronic respiratory symptoms such as cough, fatigue, fever, diaphoresis, arthralgia, and weight loss [1–4]. During the progression of the disease, there may be loss of lung function in 10% of those affected [6–8].

ICEP has a very low incidence in the pediatric population [9, 10]. In adults, it affects women: men at a 2:1 ratio, is found in patients with a history of asthma in up to 75% of cases, and can accompany severe asthma [2–4]. On chest X-ray and computed tomography (CT), alveolar and interstitial infiltrates are observed with a predominance of the bilateral, subpleural alveolar pattern in the upper lobes, which may be migratory. The infiltrates are distributed in the periphery, yielding an image that has been called the photographic negative of pulmonary edema; they can appear in superposition with an organized pneumonia or predominate with a ground-glass appearance [2, 11, 12].

Oral steroids (OSs) for 3 to 6 months are the standard management of ICEP [5, 13]. There is generally a favorable response with remission of symptoms and resolution of radiographic findings. Some patients may require longer management, of 1 to 3 years, at which time side effects of the OS can arise [5, 6, 13]. In addition, relapses of the disease occur in up to 50% of cases during the decrease or suspension of steroids, requiring restart of the drug to control the disease [6, 13].

The potential deleterious side effects of OS have made it necessary to evaluate additional treatments that allow us to control the disease and avoid complications [6, 14, 15]. Treatment with monoclonal antibodies for ICEP has been proposed based on current knowledge of severe asthma, eosinophil biology and T2 inflammation [6, 15, 16]. The first of these for severe asthma, omalizumab, binds to immunoglobulin E (IgE). The recently approved anti-IL-5 drug mepolizumab binds to interleukin-5 (IL-5), reslizumab is an intravenous drug with the same mechanism of action as mepolizumab, and benralizumab binds to the IL-5 receptor. Finally, dupilumab binds the IL-4 receptor, blocking IL-4 and IL-13 action, reducing airway eosinophilia in most patients [6, 16, 17]. All of them are useful and safe for the management of severe asthma, but their usefulness in the management of ICEP is unknown (Table 1).

**Table 1:** Monoclonal antibodies for the management of severe asthma

Monoclonal Antibody	Mechanism of action and route of administration
**Omalizumab**	Human monoclonal antibody IgG1κ, subcutaneous. It binds to free IgE by inhibiting its binding to high- and low-affinity IgE receptors (FcεRI and CD23). This reduces the expression of the aforementioned receptors in mast cells, basophils, and dendritic cells, limiting the type 2 immune response mediated by IgE. It also modulates the production of interferon-alpha (IFN-α) dendritic cells, reducing virus-induced exacerbations.
**Mepolizumab**	Humanized monoclonal antibody IgG1κ, subcutaneous. It inhibits the maturation, activation, proliferation, and recruitment of eosinophils. It binds to a specific epitope of IL-5, preventing its interaction with the IL-5 receptor (IL-5Rα).
**Reslizumab**	Humanized monoclonal antibody IgG4κ, intravenous. It inhibits the maturation, activation, proliferation, and recruitment of eosinophils. It binds to IL-5, preventing its binding to IL-5Rα. It’s in vitro affinity for IL-5 and its ability to suppress proliferation is greater than that of mepolizumab.
**Benralizumab**	Humanized monoclonal antibody IgG1κ, subcutaneous. It binds to the alpha subunit of the receptor (IL-5Rα) avoiding the transduction of eosinophil survival signals. In addition, there is an amplified apoptosis mechanism induced by the activation of the FcyRIIIa (CD16a) receptor, mediated by natural killer cells and macrophages through a process called antibody-dependent cellular cytotoxicity. It reduces eosinophils and basophils.
**Dupilumab**	Human monoclonal antibody IgG4, subcutaneous. It binds to the alpha subunit of the IL-4 receptor (IL-4Rα) shared by IL-4 and IL-13, thereby inhibiting the type 2 immune response. Simultaneous receptor blockade occurs in hematopoietic and nonhematopoietic cells.

Modified from Agache et al.

In this scoping review, the extent of the evidence for the use of monoclonal antibodies in patients with ICEP is evaluated, the types of studies on this subject are defined, and their main findings are summarized.

## Materials and methods

### Search strategy

The electronic search was carried out without time limits for English-language articles in the EMBASE, OVID, PubMed, Scopus, and LILACS databases. The terms used for the search were ((“Pulmonary Eosinophilia”) OR (“Carrington syndrome”)) AND (“Antibodies, Monoclonal, Humanized” OR “Antibodies, Monoclonal, Murine-Derived” OR “Antibodies, Monoclonal”). In conducting this scoping review, the parameters proposed by Arksey and O’Malley, 2005 [18] were considered.

### Selection of studies and data collection

The search was carried out by two independent observers (IC and AM). The selected articles were reviewed by both of them, who screened each article for the inclusion criteria. A third independent reviewer (AG) resolved any eligibility disagreements.

### Eligibility criteria

Analytical and observational studies were included. These could be cohort studies and case reports published in journals, academic sessions of poster presentations, and letters to the editor. They had to address pediatric and adult patients with the diagnosis of ICEP who required management with monoclonal antibodies. Annexes and supplementary materials were also included. Articles that did not fall under this topic, literature reviews, duplicate publications, and articles without full text available were excluded.

### Study selection and data abstraction

For the case reports, the observers recorded details about the publication (title, first author, date of publication), details about the participants (number of patients included, demographic characteristics), the biological studied, the duration of follow-up, outcomes, and any adverse events reported. Of the identified studies and case series, the study design, follow-up, number of participants, variables analyzed, intervention, outcomes and results were recorded. Tables 2, 3, 4, 5 and 6 summarize the studies and case reports recorded.

**Table 2:** Studies and case series of monoclonal antibodies for the management of ICEP

Study	Design	Follow-up	Participants	Intervention	Outcomes	Excluded	Variables	Results
Brenard et al. 2020 [19].	Multicenter, open-label, retrospective study (real-life study)	Median follow-up 9 months from diagnosis to start of mepolizumab. Median follow-up after initiation of mepolizumab 9 months (6–12 m)	12 patients, 2 excluded. Total 10 (5 men, 5 women)	Mepolizumab 100 mg every 4 weeks (6 patients: approved dose for severe asthma; 4 patients: 300 mg every 4 weeks/EGPA and hypereosinophilic syndrome)	- Annual relapse rate - Use of systemic corticosteroids before and after the start of mepolizumab - Lung lesions, evaluated by CT before starting the biological and at the last follow-up while on the biological	-Patients with eosinophilia related to JAK1 inhibitors and PDGFRα- FIP1L1. -Multiorgan involvement (heart, skin) -Infections -Findings secondary to other medications	Peripheral eosinophilia, BAL, lung function, CT, Comorbidities, and exposures Relapse: symptoms (cough, dyspnea) with increased pulmonary eosinophils, radiographic or CT changes in the absence of infection. -All had at least 1 relapse before mepolizumab.	No significant differences between doses of mepolizumab. Annual relapse rate reduction. Decrease in eosinophils at 3 months. 7 of 8 evaluated by CT had complete resolution, after the 6th month of mepolizumab. Two patients were evaluated with radiography at 6 months and no alterations were found. OS dose reduction in 9 patients at the 3rd month. At the 6th month only one patient still took OS, but in low doses due to tolerance of the decline. No patient had secondary events in the study.
Askin & Morris 2021 [20].	Retrospective Case Series	Not described	53 patients, 12 received biological.	Mepolizumab Benralizumab They do not describe doses, nor do they describe treatment allocation	Improvement in radiological, clinical, psychological changes; relapses; decrease or suspension of corticosteroid	Hypereosinophilic syndrome, patients who did not have a BAL study, or a report of eosinophils in BAL < 20%	Not described	All had sustained improvement in radiological and clinical changes, including lung function and psychological field. There were no relapses. OS decreased in all patients. No patient had serious secondary events.
Tashiro et al. 2022 [21].	Retrospective Case Series	Patient 1:8 months Patient 2:4 months Patient 3:19 months Patient 4:17 months	30 patients with the diagnosis, 12 relapses (6 had > 2 relapses), 4 received anti-IL5 biologic	2 men aged 68 and 74: Mepolizumab 100 mg every 4 weeks. 2 women aged 67 and 37: Benralizumab 30 mg every 4 weeks × 3 doses, then 30 mg every 8 weeks	Relapses, decrease in eosinophils, withdrawal, or reduction of corticosteroid dose	Findings due to drugs, mycoses, parasitic diseases, EGPA, ABPA, and other systemic diseases.	Patients with T_0_ > 37.5 °C, cough > 2 weeks, pulmonary infiltrates on CT, eosinophilia > 1000, BAL eosinophils > 25%, improvement of changes after initiation of steroids	1. Patient with type 2 diabetes, without relapses after starting benralizumab. Improvement at 4 weeks in FEV1 and absence of eosinophils. Systemic corticosteroid was withdrawn. 2. Patient without comorbidities, without relapses after starting benralizumab. Absence of eosinophils, there is no complete withdrawal of systemic corticosteroid, but dose reduction continues. 3. Patient with hypertension, hyperlipidemia, and heart failure, without relapses after the start of mepolizumab, at 4 weeks eosinophil reduction. Systemic corticosteroid was withdrawn. 4. Patient with type 2 diabetes mellitus, without relapses after the start of mepolizumab, at 4 weeks eosinophil reduction. Systemic corticosteroid was withdrawn.

**Table 3:** Case reports of benralizumab for the management of ICEP

Doses	Number of participants	Duration of follow-up after biological	Age/sex/Comorbidities	Outcomes							Case report reference
				Respiratory symptoms	Relapses	Systemic corticosteroid	Radiological findings	Lung function	Quality of life	Adverse events	
30 mg every 4 weeks for 3 doses then every 8 weeks	1	8 months	Male, 16 years old. Cortico dependent evolution, weight gain, muscle weakness	Symptom improvement immediately after onset	No relapses after initiation	OS dose was reduced after 4 months, then stopped	Not described	Improvement after the start ^a^	Improvement after the start ^a^	No adverse events during handling	David, Y. et al. 2021 [22].
Single dose, no dose mentioned	1	9 months	Female, 43 years old. Smoker: 10 cigarettes/day. Relapse, refused restarting systemic corticosteroids	Improvement after administration in the first week	None after start	They did not indicate him, only biological	Significant improvement at one month	Not described	Not described	Not described	Izumo et al. 2020 [23].
30 mg single dose	1	4 months	Female, 58 years old. Severe asthma, eosinophilic otitis media. Frequent relapses. Did not undergo LBA	Symptom improvement at 2 weeks, asymptomatic at 8 weeks	None after start	Not indicated, the biological was started	Improvement at 2 weeks with complete resolution at 8 weeks	FENO reduction at 2 weeks (102 vs. 82)	Not described	Not described	Isomoto et al. 2020 [24].
30 mg every 4 weeks, for 3 doses	1	3 months	Female, 31 years old. Did not undergo LBA. Did not accept treatment with a systemic steroid	Improvement at 2 weeks	None after start	They did not indicate him, only biological	Normalization at 5 weeks	Not described	Not described	Not described	Izhakian et al. 2022 [25].
30 mg every 4 weeks for 3 doses, then 30 mg every 8 weeks	1	24 months	Female, 52 years old. Asthma: multiple relapses	Complete improvement with ACT at 2 months	None after initiation of treatment. Sustained clinical and radiological improvement at 6, 9, and 24 months of follow-up	Allows reduction of corticosteroid 2 months after starting treatment	Complete resolution at 7 months (TC)	Normalization 5 months after treatment	Not described	No adverse events during handling	Angeletti et al. 2022 [26].
Do not describe	1	Not described	Female, 83 years old. Severe asthma, eosinophilic bronchiolitis	Improvement after the start ^a^	None after start ^a^	Tolerated descent and withdrawal ^a^	Resolution after treatment ^a^	Improvement after the start ^a^	Improvement ^a^	No adverse events during handling	Takano et al. 2021 [27].
30 mg every 4 weeks for 3 doses, then 30 mg every 8 weeks	1	30 months	Male, 57 years old. Asthma, allergic rhinitis, eczema, anxiety, depression, prostate hypertrophy. Cortico dependence, type 2 diabetes, osteopenia	Improvement after the start. ^a^	None after the start. Continued with management without relapses	Reduction at 3 weeks	Resolution after treatment ^a^	Improvement after treatment ^a^	Not described	Not described	Ricketti & Ricketti 2021 [28].
30 mg every 4 weeks	1	12 months	Female, 70 years old. Severe asthma	Improvement a week	None after start	Not indicated. The biological was started	Improvement after a week, with complete resolution at 4 weeks	Improvement at 4 weeks after handling	Not described	No adverse events during handling	Yazawa et al. 2021 [29].
Not mentioned	1	Not described	Male, 57 years old. Smoker, 20 pack-years. Cortico dependent	Improvement after the start ^a^	Not described	Not described	Not described	Not described	Not described	Not described	Braga et al. 2020 [30].
30 mg every 4 weeks	1	Not described	Female, 31 years old. Steroid-induced diabetes, Cushing syndrome, oxygen requirement	Improvement within a week (less need for oxygen)	Not described	Not described	Not described	Not described	Not described	Not described	Garcia-Saucedo et al. 2019 [31].

^a^Does not report how long it started

**Table 4:** Case reports of mepolizumab for the management of ICEP

Doses	Number of participants	Duration of follow-up after biological	Age/sex/Comorbidities	Outcomes							Case report reference
				Respiratory symptoms	Relapses	Systemic corticosteroid	Radiological findings	Lung function	Quality of life	Adverse events	
100 mg every 4 weeks. Benralizumab 30 mg every 4 weeks for 3 doses, then every 8 weeks	1	24 months Not described	Female, 58 years old. Severe asthma	Significant improvement at 4 months with ACT in control range After relapse improvement, keeping ACT in control range	None after start 1 relapse with onset	Tolerated slow decline with suspension at 12 months. Tolerated descent and suspension after relapse	Resolution at 4 months It had no alterations	Improvement at 4 months It had no alterations	Not described Not described	No adverse events during handling No adverse events during handling	Shimizu et al. 2020 [32].
100 mg every 4 weeks for 6 months, then Reslizumab 3 mg/kg every 4 weeks	1	14 months	Female, 42 years old. Type 2 diabetes, smoker 28 pack-years, frequent relapses, Cushingoid facies, acne	Improvement at 2 weeks	None after initiation of mepolizumab, 1 relapse with Reslizumab	Dose reduction at 2 weeks, suspension at 2 months	Improvement after the start ^a^	Not described	Not described	Injection site reaction and mild anaphylaxis with mepolizumab at 6 months was discontinued. No events with Reslizumab	Sarkis et al. 2020 [33].
100 mg single dose	1	Not described	Female, 57 years old. Severe asthma, type I diabetes mellitus required bronchial thermoplasty	Improvement after treatment ^a^	None after start ^a^	Suspended, followed by starting the biological	Resolution after treatment ^a^	Not described	Not described	Not described	Otoshi et al. 2020 [34].
100 mg every 4 weeks	1	7 months	Male, 45 years old. Asthma, cortico dependent	Improvement after the start ^a^	None after start	Tolerated descent and withdrawal ^a^	Not described	Not described	Not described	It caused the suspension of the biological, but they do not expand or characterize it	McKillion et al. 2021 [35].
Every 4 weeks, no dose mentioned	1	9 months	Female, 38 years old. Major depression and anxiety. Insomnia and weight gain	Improvement after initiation being significant at month 8	No relapses after initiation	Allows descent and suspension 5 months after initiation	Not described	Not described	Not described	Not described	Cyca et al. 2022 [36].
100 mg every 4 weeks	2	15 months 6 months	Case 1: Female, 56 years old. Type 2 Diabetes Mellitus Case 2: Male, 48 years old. Asthma, rhinitis, type 2 diabetes mellitus, depression	Improvement after the start ^a^	None after start	Tolerated descent and withdrawal ^a^	Normalization in X-ray and CT in both cases ^a^	Case 1: FEV1 from 55% pretreatment to 85% posttreatment ^a^ Case 2: FEV1 from 60% pretreatment to 72% posttreatment ^a^	Not described	Not described	Eldaabossi et al. 2021 [37].
100 m every 4 weeks	1	12 months	Female, 66 years old. Corticodependent asthma, atrial fibrillation, oxygen demanding, received management with Omalizumab without improvement	Improvement after the start, they withdraw oxygen ^a^	None after start ^a^	Tolerated descent and withdrawal ^a^	Not described	Not described	Not described	Not described	Benipal et al. 2021 [38].
100 mg every 4 weeks for 14 doses then every 8 weeks 100 mg every 4 weeks for 12 doses then every 8 weeks	2	36 months 24 months	Case 1: Male, 24 years old. Asthma No BAL or biopsy for diagnosis. Case 2: Female, 26 years old. Asthma Corticodependence	Improvement after the start ^a^ Improvement after the start ^a^	None after start None after start	Tolerated decline with suspension at 10 months. Tolerated decline with suspension at 10 months	Complete resolution at 14 months, sustained at 36 months. Complete resolution at 12 months, sustained at 24 months	Improvement at 14 months, sustained at 36 months. Improvement at 12 months, sustained for 24 months	Not described Not described	No adverse events during handling No adverse events during handling	Sato et al. 2021 [39].
300 mg every 4 weeks	1	18 months	Female, 55 years old. Asthma, atopic dermatitis, rhinitis, anxiety. Corticosteroid intolerance	Improvement after treatment ^a^	None after start ^a^	Allows corticosteroid decrease	Not described	Not described	Not described	No adverse effects r	Kisling et al. 2020 [40].
No dose indicated	1	Not described	Female, 47 years old. Corticodependent	Improvement after treatment ^a^	None after start ^a^	Tolerated reduction and withdrawal ^a^	Not described	Not described	Not described	Not described	Askin et al. 2020 [41].
100 mg every 4 weeks	1	10 months	Female, 59 years old. Asthma HTN, hyperglycemia, osteoporosis	Improvement after 3 months with ACT in adequate control	None after start	Not indicated. The biological was started	Resolution 3 months after starting treatment	Improvement after treatment _a_	Not described	No adverse events during handling	Ciuffreda et al. 2020 [42].
300 mg every 4 weeks	1	Not described	Female, 55 years old. Asthma, anxiety, and steroid-related hallucinations	Improvement after the start _a_	Not described	Tolerated descent _a_	Not described	Not described	Not described	Not described	Jones et al. 2019 [43].
100 mg every 4 weeks	1	12 months	Female, 47 years old. Asthma, rhinitis. 3 relapses (2 in less than 6 weeks	Improvement after the start _a_	None after start	Tolerated descent and withdrawal _a_	Not described	Not described	Not described	Not described	McInnis et al. 2019 [44].
100 mg every 4 weeks	2	2 months	Case 1: Female, 54 years old. Asthma, rhinitis, chronic rhinitis with nasal polyps, idiopathic thrombocytopenic purpura. Diagnosed in 2012. Two relapses Case 2: Female, 21 years old. Nonallergic asthma, rhinitis, sensorineural hearing loss. Diagnosed in 2015. Three relapses	Significant improvement 8 weeks after starting the biological	None after start	Case 1: Tolerated decline but is maintained by hematological comorbidity. Case 2: Tolerated decrease to 5 mg/alternate days prednisolone	Not described	Case 1: pretreatment FENO > 300 ppb, post treatment 157 ppb Case 2: not described	Not described	Not described	Mendes et al. 2019 [45].
Not describe doses.	1	4 months	Female, 60 years old. Severe asthma, eosinophilic bronchiolitis	Improvement after the start _a_	No relapses after initiation	Not indicated, the biological was started	Improvement _a_	Improvement _a_	Not described	Not described	Tomyo & Sugimoto 2019 [46].
They do not describe doses. Vedolizumab continued	1	6 months	Female, 49 years old. HBP, ulcerative colitis in management with vedolizumab and nodular prurigo, ex-smoker. Diagnosis by lung biopsy. Corticodependence	Significant improvement 7 days after onset	No relapses after initiation	Allows descent	Significant improvement at 6 months	Not described	Not described	No adverse events during handling	Lawrence et al. 2019 [47].
100 mg every 4 weeks	1	13 months	Male, 65 years old. Asthma relapses.	Resolution at 4 weeks	None after the start, continued with management	Not indicated. It was switched to the biological	Resolution 3 months after starting treatment	Not described	Not described	No adverse events during handling	To et al. 2018 [48].

^a^Does not report how long it started

**Table 5:** Case reports of omalizumab for the management of ICEP

Doses	Number of participants	Duration of follow-up after biological	Age/sex/Comorbidities	Outcomes							Case report reference
				Respiratory symptoms	Relapses	Systemic corticosteroid	Radiological findings	Lung function	Quality of life	Adverse events	
150 mg every 4 weeks. Mepolizumab 100 mg every 4 weeks	1	12 months 18 months	Female, 48 years old. Asthma, chronic rhinosinusitis without polyps. Diagnosis by biopsy	Partial improvement with Omalizumab. No symptoms after 4 weeks of initiation of mepolizumab	None after start	Reduction of the dose, but allows to suspend it. Allows reduction and suspension 24 months after starting mepolizumab	No improvement during the 12 months with Omalizumab Complete resolution 14 months after the start of mepolizumab	No improvement during the 12 months with Omalizumab Improvement 3 months after starting mepolizumab, normalization at 12 months	Not described	No adverse events during handling	Lin et al. 2019 [49].
300 mg every 2 weeks for 9 months. Dose per total IgE level. 225 mg every 2 weeks for 3 months. Dose per total IgE level. Then, 150 every 2 weeks for 2 months	2	33 months 20 months	Case 1: Male, 17 years old. Asthma, sensitization to aeroallergens. Case 2: Male, 19 years old. Asthma, sensitization to aeroallergens	Improvement after the start, being complete at 9 months. Complete improvement at one month	No relapses after initiation No relapses after initiation	Allows descent and suspension 5 months after initiation. Allows descent with suspension one month from the start	Improvement at 5 months Improvement at 5 months	Not described Not described	Not described Not described	No adverse events during handling No adverse events during handling	Shin et al. 2012 [50].
300 mg every 2 weeks. Dose per total IgE level 429 IU/mL. Treatment for 18 months. Restart after relapse at the same dose for 24 months	1	69 months	Female, 68 years old. Osteoporosis, aeroallergen sensitization	Improvement after the start. ^a^ Relapse 10 months after withdrawal, with improvement a few weeks after initiation. ^a^ After stopping it, without relapses at 17 months	10 months after the withdrawal so it is restarted, without relapses 2 years after the restart	Descent after a few weeks. It requires restart after relapse, tolerating descent and withdrawal. ^a^	Complete normalization at 17 months. After relapse, normalization at 2 years after restart.	Not described	Not described	Not described	Nehme et al. 2022 [51].
Omalizumab (dose not mentioned)	1	Not described	Female, 55 years old. Allergic asthma, rhinoconjunctivitis. On lung transplant list	Improvement after the start. Withdrawal from transplant list	None after start	Withdrawal at 24 months	Not described	Not described	Not described	Not described	Laviña Soriano et al. 2017 [52].
300 mg every 4 weeks for 18 months, then decrease 50% every 6 months until discontinuation	1	45 months	Female, 36 years old. Asthma Depression and steroid amenorrhea	Improvement after the start. ^a^	None after the start. No relapses after 15 months of finishing the treatment	Tolerated descent and suspension at the 4th week	Resolution after treatment ^a^	Not described	Not described	No adverse events during handling	Kaya & Tozkoparan 2012 [53]. Domingo & Pomares 2013 [54].

^a^Does not report how long it started

**Table 6:** Case report of dupilumab for the management of ICEP

Doses	Number of participants	Duration of follow-up after biological	Age/sex/Comorbidities	Outcomes							Case report reference
				Respiratory symptoms	Relapses	Systemic corticosteroid	Radiological findings	Lung function	Quality of life	Adverse events	
Dupilumab 300 mg every 2 weeks for 6 months	1	12 months	Female, 11 years old. No response to antibiotic management, systemic steroids, or cyclosporine	Improvement at 2 weeks	None after start	Tolerated withdrawal and decrease in cyclosporine	Improvement at 2 weeks	Not described	Not described	No adverse events during handling	Fowler 2020 [55].

### Statistical analysis

Normally distributed dichotomous variables are reported as n (%) and continuous ones as median (IQR) or mean (SD). Statistical analyses were performed with Stata® 14.0 (Stata Corp., 2014, College Station, TX, USA).

## Results

**Fig. 1:**
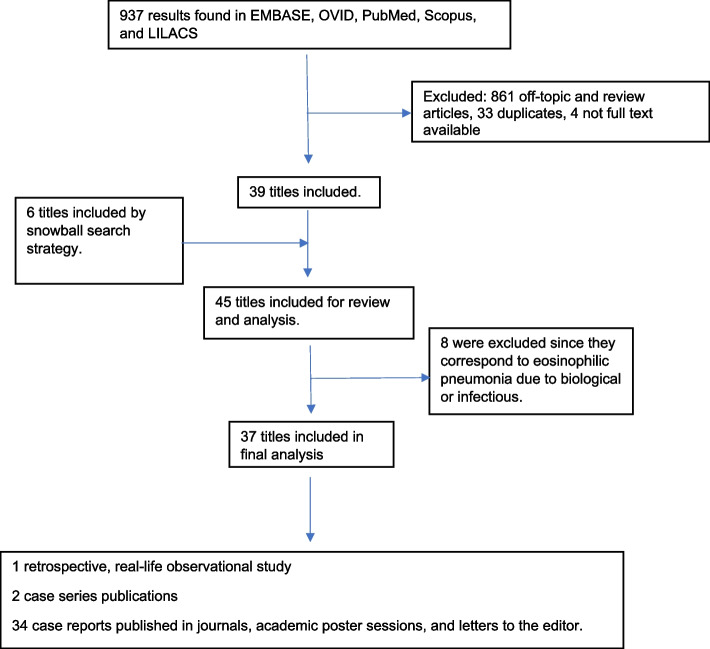
PRISMA flow diagram

The search strategy yielded 937 results. After applying the exclusion criteria, 39 manuscripts were obtained, to which six articles were added that were found in the bibliographic references. More detailed analysis excluded eight articles, leaving 37 titles for the final analysis: a retrospective observational study done in real time, two case series, and 34 case reports. One patient was described by two articles, a first report and a follow-up report. The flow chart in Fig. 1 shows the selection process in detail. Studies published up to December 2022 were included.

A total of 63 patients received monoclonal antibody management, but one publication did not describe how the allocation was done for the 12 patients who received a biological. The median age was 49 years (57.5–31), and 70.5% were women. Regarding the assigned treatment, 31 patients received mepolizumab, 14 received benralizumab, five received omalizumab and only one received dupilumab. With mepolizumab, some patients were given the approved dose for granulomatosis with polyangiitis (EGPA) and others were given the approved dose for severe eosinophilic asthma. There were three case reports in which patients received more than one monoclonal antibody. Two patients switched from mepolizumab to benralizumab and reslizumab respectively. The first switched because of relapse after 24 months on mepolizumab and achieved adequate control with benralizumab [32]. The second patient switched to mepolizumab due to anaphylaxis and had good tolerability and adequate disease control with reslizumab [33]. The last patient switched from omalizumab to mepolizumab after failing to achieve a clinical response after 12 months on omalizumab, and reported a complete response after 12 months on mepolizumab [49].

In the open, retrospective, real-life study conducted by Brenard et al., 10 patients were included with relapsing ICEP and did not include patients with hypereosinophilic syndrome (HES) or EGPA. Six patients received mepolizumab at a dose of 100 mg every 4 weeks, and four received 300 mg every 4 weeks. The median follow-up was 9 months after the start of mepolizumab [19]. There were no significant differences in disease response by dosage. With both doses, a significant reduction in the annual relapse rate was observed with mepolizumab, both falling to a value of 0 [19]. In seven of eight patients evaluated by CT, complete resolution was found after the 6th month on mepolizumab. Two patients were evaluated with a chest radiograph at 6 months, and no alterations were found. The decrease in OS dose began after the 3rd month of management, and only one patient did not tolerate the decrease [19]. At the 6th month of management, only one patient still needed the OS, but at low doses due to adequate tolerance of the low-dose OS. No patient had secondary events in the study [19].

In the retrospective case series by Askin & Morris, which included 53 patients, 12 patients were treated with mepolizumab or benralizumab. They included patients with a diagnosis of ICEP and excluded those with HES, those without a BAL study or those with a reported BAL eosinophil count < 20%. All cases were either relapsing or refractory to glucocorticosteroids after weaning trials [20]. All patients achieved sustained clinical improvement, including lung function and psychological status, as well as in radiological findings. There were no relapses, all patients lowered their OS dosage, and no serious secondary events were reported. The shortcoming of this case series was that it did not describe the treatment allocation, the duration of follow-up, or the dose used [20].

In the retrospective case series by Tashiro et al., all of the patients included in the study fulfilled the diagnostic criteria for ICEP. Patients with drug-induced eosinophilia, mycoses, parasitic diseases, EGPA, bronchopulmonary aspergillosis, HES, and patients with satisfactory response to OS without relapse were excluded. Twelve of the 30 patients relapsed while OS was declining, and six of them had more than two relapses. Four patients were assigned to treatment with monoclonal antibodies, of whom only one did not have any comorbidities [21]. Two patients received mepolizumab at a dose of 100 mg every 4 weeks, and two patients received benralizumab at 30 mg every 4 weeks for three doses, followed by 30 mg every 8 weeks. The two patients who received benralizumab were observed 4 and 8 months after the initiation of the monoclonal antibody [21]. In both, the absence of symptoms was observed at 4 weeks; one tolerated the decrease in OS, while the other tolerated full withdrawal. The absence of eosinophils was also observed at 4 weeks, and no adverse events were reported. The patients who received mepolizumab both stopped the OS, with a reduction in peripheral eosinophilia and clinical improvement at 4 weeks. The follow-up time after the monoclonal antibody was 19 and 17 months [21] (Table 2).

Of the case reports, 19 patients received management with mepolizumab, 12 with benralizumab, five with omalizumab and one with dupilumab. There were three patients under 18 years of age; one of them, an 11-year-old female, was the only one to receive dupilumab and had a clinical and radiological response at 2 weeks, tolerating withdrawal of OS and decreased cyclosporine. They reported no adverse effects of treatment at the 12-month follow-up [55]. Another of them was a 16-year-old male patient who received benralizumab with an 8-month follow-up from the start of the monoclonal antibody. He had immediate clinical improvement, allowing reduction and suspension of OS, evolving without relapses and with improvement in lung function [22]. The third, 17-year-old patient received omalizumab, with an observation period of 33 months from the start of the monoclonal antibody. After treatment for 9 months, there was an adequate response, with improvement of symptoms, no relapses during the follow-up period, and radiological improvement. No adverse events reported during handling [50].

The 34 remaining patients described in the case reports were adults, many of whom had comorbidities such as diabetes, hypertension, and anxiety disorders, and some reported cortico-dependent disease and secondary events derived from the management of OS. In general, an adequate response was observed, with improvement of respiratory symptoms, control of relapses, and achievement of reduction and later cessation of OS. All the reports mentioned the response of the respiratory symptoms, only two reports did not record relapses during treatment with the monoclonal antibody, and one did not mention whether the OS was decreased or suspended. The majority reported a decrease and cessation of OS after starting biological testing, but the vast majority of reports did not indicate how long this was achieved, nor did they mention using any established guideline to reduce OS.

Sixteen case reports evaluated lung function; reporting improvement compared to before the monoclonal antibody. The response time of the symptoms was variable but favorable. In general, the response began between the first week and 9 months after starting management. Eight reports did not mention the dose of the monoclonal antibody used in the management of patients.

There were two reports of treatment with a single dose of benralizumab and one with a single dose of mepolizumab. In the latter three cases, symptoms improved with resolution of relapses and cessation of OS. Two reports of patients treated with single-dose benralizumab reported adequate responses after 4 and 9 months of treatment. However, the report on single-dose mepolizumab did not record the duration of follow-up [23, 24, 34].

Twelve case reports did not report tomographic or radiological changes after treatment, and seven reports did not mention the follow-up time after the initiation of the monoclonal antibody. None of the case reports, case series, or real-life studies evaluated quality of life.

Regarding the secondary effects of the treatment, the majority reported that no events were seen during the treatment, but 17 reports did not mention whether any occurred during treatment. There were only two reports of adverse events during treatment. Sarkis et al. described a local reaction with mild anaphylaxis during management with mepolizumab, which led to a switch to reslizumab [33]. In the case report by McKillion et al., they indicated an unspecific reaction that forced the discontinuation of mepolizumab, but the reaction was not described [35].

## Discussion

Although ICEP is a rare disease, when it occurs it brings a 50% risk of relapses and a long-term need for steroids. Patients are at risk of developing complications derived from prolonged management with OS. From this scoping review, it can be inferred that monoclonal antibodies approved for severe asthma could be useful for the control of the disease, but the extent of the evidence is limited and is composed mostly of case reports and case series, though most of the results show a good response. Biological drugs seem to be a safer option to control relapses of ICEP, allowing lowering/suspension of OS and sometimes replacing the OS in patients with OS intolerance, patients with significant comorbidities, and patients who have already developed adverse events.

For omalizumab, the little evidence available is provided by case reports that have had a longer follow-up and have indicated a profile of safety and effectiveness. Omalizumab was the first biological to be approved for severe asthma.

Anti-IL-5 therapy is more recent, and although the follow-up period in most case reports is shorter, there is more information available about mepolizumab, including an observational, retrospective, real-life study, which supports its use for ICEP. The mepolizumab dose used to treat severe asthma appears to be as effective as the dose used to treat EGPA.

Mepolizumab-based treatments for ICEP show that a large majority of patients are free of symptoms after one year of treatment, and some reports show that patients are free of symptoms for up to 36 months. As a result, treatment would generally not last longer than that. However, it is important to assess each case individually. Benralizumab showed similar results with only one case being resolved after 30 months. For omalizumab, follow-up periods were longer than 24 months in most reports, and some reported relapses after discontinuation before this period, in some cases requiring a switch to another monoclonal antibody. This suggests that the duration of treatment with omalizumab may be longer than with anti-IL-5 therapy.

Benralizumab seems to be effective, and it seems that it generates a rapid decrease in eosinophils for up to 8 weeks, so authors have tried to space its doses or even give a single dose. This scoping review found no data suggesting superiority of benralizumab in treating ICEP. Two reports evaluated single-dose treatment with benralizumab and reported good control at 4 and 9 months of follow-up. The only report of single-dose treatment with mepolizumab did not report the length of follow-up. There were also no real-life studies of benralizumab for ICEP.

Reslizumab seems to have a similar effect as mepolizumab, but reports on it are even rarer, and none have evaluated it as a first-line biologic. In cases of immediate hypersensitivity to mepolizumab, reslizumab may be a safe alternative.

Dupilumab has only been used in one pediatric patient, who had a good response [55]. Increased peripheral eosinophils have been reported in patients receiving dupilumab. However, these have been shown to be transient and have not been associated with reduced efficacy of dupilumab. After week 16 of treatment, recovery of eosinophil count has been documented. Furthermore, the development of symptoms associated with increased peripheral eosinophils is rare [56]. For this reason, we believe that dupilumab is unlikely to be less effective in patients with ICEP. However, as we were selecting manuscripts and carrying out the detailed analysis, we found some case reports that have linked it as a cause of ICEP [57–60].

The findings of this review suggest that anti-IL-5 and omalizumab could be safe for the management of patients with ICEP. No adverse events of any severity were reported in the real-life study or in the case series. Only two case reports described adverse events with mepolizumab, which caused biological changes or the suspension of treatment, but no deaths or other complications were reported.

The findings of this review are not surprising, since management with biologics is recent and ICEP is a low-frequency entity. This study has limitations arising from the quality of the included studies, as they were all observational studies and case series, so its interpretation should be carried out taking into account these limitations. Given the characteristics of the included studies, it is not feasible to assess their quality. Furthermore, this makes it impossible to extrapolate the results to all patients with ICEP.

Research in this field needs to be expanded. There need to be more cohort studies or case-series studies in real-world settings, where the impact on the quality of life of the patients is considered and the follow-up time of the patients after the start of the evaluated drug is clearly described.

## Conclusion

The extent of the evidence for management with monoclonal antibodies (omalizumab, mepolizumab, benralizumab, reslizumab) is limited but could be promising for cases of ICEP with frequent relapses and cases where the use of corticosteroids is contraindicated. The extent of the evidence for management with dupilumab is even more limited. Well-designed studies that evaluate quality of life and outcomes during a clear follow-up period are needed. To our knowledge, this is the first scoping review of the literature detailing the extent of the evidence for the management of ICEP with monoclonal antibodies.

## Data Availability

Extracted information and synthesized data is available on reasonable request to the corresponding author. In addition, the protocol of this study is submitted to *“JMIR Preprint”* with the reference number 48394-758611-1-SM.docx 2023-04-21.

## Abbreviations

ICEP: Idiopathic chronic eosinophilic pneumonia
AEL: Acute eosinophilic pneumonia
OS: Oral steroid
BAL: Bronchoalveolar lavage
CT: Computed tomography
IgE: Immunoglobulin E
IgG1: Immunoglobulin G subclass 1
IgG4: Immunoglobulin G subclass 4
IL-5: Interleukin-5
IL-5Rα: Interleukin-5 receptor
IL-5Rα: IL-5 receptor alpha subunit
FcyRIIIa/CD16a: Low-affinity immunoglobulin gamma Fc receptor region III-A
FcεRI: High affinity IgE receptor
CD23: low affinity IgE receptor
IL-4: Interleukin-4
IL-4Rα: IL-4 receptor alpha subunit
ABPA: Allergic bronchopulmonary aspergillosis
EGPA: Eosinophilic granulomatosis with polyangiitis
HT: Arterial hypertension
ACT: Asthma control test
FENO: Exhaled nitric oxide
FEV1/FEV1: Forced expiratory volume in the first second
JAK 1: Janus kinases type 1
FIP1L1-PDGFRA: Fip1-like 1-platelet-derived growth factor receptor alpha
